# Understanding the impact of perineal reconstruction after extended margin cancer surgery on longer-term quality of life, morbidity and health economic outcomes: protocol for the UK REMACS observational cohort study

**DOI:** 10.1136/bmjopen-2026-124218

**Published:** 2026-09-29

**Authors:** Malcolm A West, Kashuf Khan, Charles T West, Zoe Li, Elias Brown, Samantha Sodergren, Helen Moyses, Sam A Rose, Errol Bignall, Layla Stephen, Sharon A Applin, Keith Cooper, Inês Souto Riberio, Lynn Calman, Alex H Mirnezami

**Affiliations:** 1 Cancer Sciences, University of Southampton Faculty of Medicine, Southampton, UK; 2 NIHR Southampton Biomedical Research Centre, University Hospitals Southampton NHS Foundation Trust, Southampton, UK; 3 Department of Plastic Surgery, University of Swansea, Swansea, UK; 4 Centre for Psychosocial Research in Cancer, University of Southampton, Southampton, UK; 5 Bowel Research UK, London, UK; 6 Communities First charity, London, UK; 7 PLANETS Cancer Charity, Southampton, UK; 8 Southampton Health Technology Assessments Centre, University of Southampton, Southampton, UK

**Keywords:** Colorectal surgery, QUALITATIVE RESEARCH, HEALTH ECONOMICS

## Abstract

**Introduction:**

Advanced and recurrent pelvic cancers pose significant public health challenges due to their invasive nature, affecting pelvic structures like the pelvic floor, other organs and the perineum. Treatment involves chemoradiotherapy followed by extended margin cancer surgery (EMCS) to achieve cancer cure. EMCS results in an empty pelvis prone to major complications like collections, abscesses, bowel obstruction, fistulae and perineal wound breakdown. Known as the empty pelvis syndrome (EPS), this affects >50% of patients. EPS leads to poor quality of life (QoL), reduced return to function and significant financial burden to patients and healthcare services. Perineal reconstruction using myocutaneous flaps or biological meshes may mitigate these complications, but their longer-term clinical outcomes and effectiveness are not well-documented. This study addresses key unmet needs and aligns with strategic healthcare priorities.

The Reconstruction after Extended Margin Cancer Surgery (REMACS) study aims to assess the effects of EMCS and perineal reconstruction on QoL, complications and health economic utilisation up to 12 months post-surgery.

Objectives include:

a. Providing real-world data on 12-month QoL and complications to aid shared decision-making consultations regarding reconstructive techniques post-ECMS.

b. Informing clinical practice and patient expectations, improving communication and reducing regret throughout the initial stages of cancer survivorship.

c. Evaluating economic impacts on patients and healthcare settings, optimising resource utilisation.

d. Uncovering hidden treatment toxicities post-ECMS.

**Methods and Analysis:**

Design: Multi-centre observational cohort study.

Participants: 236 patients undergoing EMCS with perineal reconstruction in tertiary cancer centres, ensuring diverse representation.

Data Collection: Patients will complete QoL (European Organisation for Research and Treatment of Cancer Quality of Life Questionnaire-Core 30 (EORTC-QLQ-C30), EuroQol-5 Dimension 5-Level Questionnaire (EQ-5D-5L), Locally Recurrent Rectal Cancer-QoL (LRRC-QoL)) and Decision-Regret questionnaires pre-surgery and 3, 6, 12 months post-surgery. Patient economic impact (COST, patient employment, health resource utilisation), quality-adjusted life years, incremental cost-effectiveness ratios (EQ-5D-5L) and healthcare economic impact will be recorded over 12 months after surgery. Complications (Clavien-Dindo) and pathology outcomes. 5-year survival and cancer recurrence will also be recorded as part of standard of care. Purposefully selected patients (n=30) representing different cancer types and from different geographical regions in the UK, ensuring the inclusion of different ethnic and under-represented minority backgrounds (ie, sexual and gender minorities and those from low socio-economic groups) will be interviewed at 3 and 12 months post-surgery. Patient experiences including preparedness, complex decision-making, complications, regret and hidden cancer treatment toxicities will be explored.

Analyses: EORTC-QLQ-C30 global health scores at 12 months will be compared between groups (primary outcome: flap versus mesh reconstruction). Descriptive statistics, causal inference techniques and regression modelling will be used to account for allocation bias and heterogeneity. Propensity scoring will weight cases and causal inference techniques will be used to visualise and understand confounding effects. Cost-utility economic analyses will compare reconstructive techniques. Longitudinal interview analyses will use constant comparative methods using reflexive thematic analysis.

**Ethics and Dissemination:**

REMACS is approved by HRA REC (22/NE/0032). The study is funded by Penguins Against Cancer (Registered Charity Number 1159905), Bowel Research UK (BRUK SG 23019), PLANETS Cancer Charity (Registered Charity Number 1175572) and an NIHR Research for Patient Benefit Grant (NIHR207881).

This study will benefit patients internationally by providing real-world outcomes to improve shared decision-making around complex consultations, informing patient expectations and clinical outcomes for QoL, complications and cost after EMCS and perineal reconstruction. Results will be disseminated to researchers, healthcare professionals, patients, carers, charities and policymakers through online platforms, media, scientific conferences and journals. This will raise awareness of late effects of cancer treatment, influence international guidelines and cancer commissioning.

**Trial Registration number:**

Clinical Trials.gov registration number—NCT05219058 and ISRCTN registry number—ISRCTN81241096.

**STRENGTHS AND LIMITATIONS OF THIS STUDY**

- This large, multicentre longitudinal cohort study captures real-world outcomes across diverse UK cancer centres, enhancing generalisability and representation of minoritised groups of patients.
- The mixed-methods design combines validated patient-reported outcome measures with qualitative interviews, enabling a comprehensive assessment of quality of life, complications and patient experience.
- Use of validated instruments and advanced analytical approaches (including propensity scoring) strengthens the robustness of outcome measurement and comparative analyses.
- As an observational study without randomisation, there remains potential for residual confounding and selection bias between perineal reconstruction groups.
- The 12-month follow-up and heterogeneity in cancer types, procedures and patient characteristics may limit interpretation of longer-term outcomes and between-group comparisons.

## Introduction

 Locally advanced pelvic cancers affect structures like the rectum, anus, gynaecological organs, bladder and prostate. Although uncommon, these cancers are devastating, with around 6000 patients treated annually in the UK.^1 2^ Additionally, 10% of patients experience locally recurrent pelvic cancers following previously curative surgery. These cancers are debilitating^2 3^ and are renowned for being one of the worst ways to die. They invade pelvic organs, musculature and the perineum (skin and soft tissues around anus and genitalia), causing severe pelvic pain, bowel urgency, recurrent sepsis, malodorous discharge and social isolation. These patients, who are increasingly younger and from ethnic minorities, undergo intensive chemotherapy, radiotherapy and major surgery.^4 5^ This current standard of care provides the best hope of cure. Without surgery, survival is around 5–7 months^6^; with chemotherapy and radiotherapy alone, extending to 12–14 months, with a 5-year survival of 0% to 5%.

Radical surgery, including extended margin cancer surgery (EMCS), comprises extralevator abdominoperineal excision (ELAPE) and pelvic exenteration (PE) and is the mainstay of surgical management for locally advanced and recurrent pelvic cancers. Advances in surgical technique have improved survival outcomes (5-year survival>57%^7 8^); however, achievement of clear surgical margins (R0 resections) is the greatest predictor of survival. Achieving R0 resections can lead to extensive surgery beyond normal surgical planes, involving multiple pelvic structures. ELAPE removes the entire pelvic floor, rectum and anus around the cancer often with radical resections involving the ischiorectal fossa fat, while PE further extends these margins, requiring bespoke *en bloc* removal of soft tissue, bone, other pelvic organs, pelvic musculature and the perineum to maximise cancer clearance.^2^ Despite improved cancer outcomes, complications remain high (59% all complications and >24% major complications), especially after neoadjuvant radiotherapy.^2^

This surgery is life-altering, removing critical pelvic structures and leaving behind a large empty space, an empty pelvis. Pelvic filling and perineal reconstruction with flap reconstruction or biological mesh reconstruction may mitigate major complications like chronic collections, wound breakdown, sinuses and fistulae.^8^ These complications give rise to a syndrome known as the ‘empty pelvis syndrome’(EPS), encountered in up to 50% of patients.^8 9^ Currently, limited, low-quality evidence with short follow-up provides little guidance to patients and clinicians on optimal reconstruction techniques.^9 10^ The empty pelvis syndrome has been recently defined as ‘a spectrum of post-exenteration complications including infected fluid collections, bowel obstruction, perineal sinus and fistulas—severity is multifactorial, likely due to radicality of resection and migration of bowel into the void generated”.^11^ Heterogeneity in outcomes reporting and the lack of robust data hamper advancements in this field.^10 12 13^

Patients with EPS complications experience worse overall outcomes.^14^ A systematic review from our group highlighted various surgical techniques to mitigate EPS complications.^9^ Biological meshes and flap reconstructions are common post-EMCS in the UK, but low-quality evidence hampers comparisons of techniques and outcomes reporting, with limited quality of life (QoL) assessments.^9 10 15^ Studies comparing flap vs mesh reconstruction in ELAPE did not investigate QoL.^16–18^ Primary perineal closure, associated with higher morbidity, is rarely used in EMCS in the UK.^10 19^ The BIOPEX study, the only Randomised Controlled Trial (RCT) comparing biological mesh with primary closure after ELAPE, excludes PE or flap reconstruction surgeries.^20^ BIOPEX-2 will investigate QoL outcomes in primary closure vs flap reconstruction, but again excluding PE surgery.^21^ Foster *et al*^22^ and the Association of Coloproctology of Great Britain and Ireland^10^ highlighted this large evidence gap in perineal reconstruction after EMCS. This coupled with the lack of long-term data and lack of consensus on effective EPS mitigation strategies underscores the need for further urgent research.

Quality of life (QoL) improves following EMCS^7 23 24^ but rarely returns to pre-diagnosis levels, especially with EPS complications.^25 26^ QoL assessments, mainly derived from small cohort studies, document wide-ranging negative impacts on physical domains after surgery.^24 27^ A recent systematic review highlighted the urgent need for QoL and functional outcomes to be addressed in future studies,^28^ with simple, visual QoL trajectories important to improve shared decision-making.^29^ Patients with pelvic cancers value QoL as much as survival, and an informed understanding of the potential impact of surgical treatment on their physical and psychosocial status is crucial for shared decision-making. Patients with EPS suffer poor long-term QoL, poor return-to-function and life-limiting complications, leading to further surgeries and prolonged hospitalisation,^30^ with significant financial burden to patients and the National Health Service (NHS).^9 30^ These aspects of cancer surgery are poorly explored. Moreover, exploring aspects of decision-regret and ‘hidden toxicities’ from cancer treatments^31^ including surgery represent a large unmet need for this patient group.

Health economic evidence post-EMCS is also limited,^20 32–35^ with some prospectively collected data after advanced pelvic cancer reconstruction showing that high-complexity PE are associated with significant costs especially if infra-levator reconstruction is carried out with biological mesh, myocutaneous flaps or a composite reconstruction. Financial toxicity, which refers to the objective financial burden and subjective financial distress experienced by patients, is a significant issue that reduces QoL. It is deemed to be a clinically relevant patient outcome, yet this subject remains under-researched. Understanding resource utilisation and hidden financial toxicities relating to cancer treatment is an important aspect to obtaining a holistic understanding of the impact on patients.

To address these unmet needs, REMACS aims to determine the effects of EMCS, specifically comparing 12-month QoL, complications and health economic utilisation between flap and mesh perineal reconstruction groups, with a predefined subgroup analyses for patients that undergo primary closure after ECMS. This research addresses key James Lind Alliance^36^ priorities for patients ‘Living With and Beyond Cancer’. It also aligns with strategic National Cancer Plan for England^37^ commitments to improving QoL, patient experience, personalisation of care and reducing variations in healthcare.^12^

This study will address key current unmet needs by:

1. Providing real-world longer-term QoL outcomes for use in shared decision-making consultations, aiding decisions on reconstructive techniques after ECMS.
2. Providing robustly collected outcomes, informing patient expectations around the late effects of perineal reconstruction complications after surgery.
3. Documenting the economic impacts on patients and the NHS, enabling better resource utilisation and uncovering hidden treatment toxicities.
4. Conducting a mixed-methods evaluation of primary and secondary outcomes to identify which endpoints will be most meaningful and valuable to patients and clinicians for use as a primary outcome for future implementation research.

REMACS specifically will provide robust outcome data to inform patient expectations and clinician decision-making, essential to improving patient care and changing international practice. Therefore, REMACS aims to determine the effects of EMCS, specifically comparing 12-month QoL, complications and health economic utilisation between perineal flap and mesh reconstruction groups. A planned additional analysis of patients undergoing primary closure after EMCS will be undertaken.

### Primary objective

To compare QoL at 12 months between flap and mesh perineal reconstruction groups after EMCS using EORTC QLQ-C30.

#### Secondary objectives

- Comparing longitudinal generic and cancer-specific QoL trajectories pre-surgery, 3, 6 and 12 months post-surgery between reconstruction groups.
- Comparing complications between reconstruction groups during the 12 months post-surgery.
- Assessing incremental cost-effectiveness ratios (costs per additional quality-adjusted life year gained) between reconstruction groups during the 12 months post-surgery.
- Understanding changes in patient health economic impact, with financial toxicity measurements (pre-surgery, 3, 6,12 month post-surgery) between reconstruction groups.
- Assessing NHS healthcare utilisation between reconstruction groups up to 12 months post-surgery.
- Understanding changes in decision-making and decisional regret between reconstruction groups.
- Comparing overall and disease-free survival at 12 months post-surgery between reconstruction groups.

## Methods and analysis

### Study design

Multicentre observational longitudinal cohort study.

### Participants and eligibility

#### Inclusion

- Male and female patients aged 18 years and over.
- Locally advanced or locally recurrent cancers arising from the rectum, uterus, ovary, cervix, anus, bladder, prostate and sarcomas.
- Patients undergoing curative intent EMCS including infralevator PE or extralevator abdomino-perineal excision.
- Patients undergoing perineal reconstruction with myocutaneous flap, biological mesh or skin closure.

#### Exclusion

- Unfit, declined or not offered curative intent surgery.
- Inter-sphincteric abdominoperineal resection only.
- Inability or unwillingness to provide informed consent.
- Inability to complete the questionnaire independently online or over the telephone with a researcher, with/without translator support.
- Patients who are prisoners in the custody of HM Prison Service or who are offenders supervised by the probation service.

### Study setting

This study will take place in secondary and tertiary care surgical centres in the UK that treat locally advanced and locally recurrent cancer patients with EMCS and offer perineal reconstruction techniques. The following centres will be recruiting to REMACS, these undertake more than 60% of all EMCS performed in the UK. Participating centres include University Hospital Southampton NHS Foundation Trust, Salisbury District Hospital, University Hospitals Dorset NHS Foundation Trust, Somerset NHS Foundation Trust, Dorset County Hospital NHS Foundation Trust, Portsmouth Hospitals University NHS Foundation Trust, Hampshire Hospitals NHS Foundation Trust, Leeds Teaching Hospitals NHS Trust, The Newcastle upon Tyne Hospitals NHS Foundation Trust, Royal Devon University Healthcare NHS Foundation Trust, Mid-Yorkshire Hospitals NHS Trust, University Hospitals Sussex NHS Foundation Trust, London North West University Healthcare NHS Trust, Swansea Bay University Health Board NHS Wales, Manchester University NHS Foundation Trust, NHS Greater Glasgow and Clyde, The Christie NHS Foundation Trust, University Hospitals of Leicester NHS Trust, Lancashire Teaching Hospitals NHS Foundation Trust and Northern Care Alliance.

These centres were selected due to their ethnic and socioeconomic diversity and their representation of diverse urban, rural and coastal communities.

The study was set up in May 2022 (pilot phase), obtained NIHR Research for Patient Benefit funding (NIHR207881) in August 2025 and is currently in its recruitment phase that is planned until February 2027, with follow-up and reporting planned until February 2028.

### Recruitment and consenting

Eligible patients will be identified by clinical and research teams and provided with a Participant Information Sheet and a verbal explanation of the study. All patients who choose to participate will complete an electronic or paper consent form.

All patients will consent for the observational component of REMACS, with patients purposefully sampled and re-consented for the qualitative component. Patients will be purposefully sampled to ensure representation across age, sex, gender, sexual orientation, ethnicity, type of cancer, operation type, education level, employment, marital status, caring responsibilities and level of social support at home.

### Data collection—observational study

Patient demographics including residential postcode, cancer characteristics and treatment details, tumour and survival outcomes will be collected from pre-operative baseline to 12 months after surgery. This includes resource utilisation data—in-hospital stay, critical care and theatre utilisation, in-hospital complications, re-admission, re-operations and morbidity up to 12 months after their index admission.

Patients will complete the following validated Patient Reported Outcome Measures (PROMs) at the pre-operative, 3 months, 6 months, and 12 month’s post-operative time points:

- EQ-5D-5L.^38^
- European Organisation for Research and Treatment of Cancer Quality of Life Questionnaire-Core 30 (EORTC-QLQ-C30) with specific cancer-type modules (see table 1).^39^
- Locally Recurrent Rectal Cancer (LRRC-QoL) (only after August 2025).^40^
- Decision regret questionnaire.^41^

**Table 1:** List of cancer-type-specific PROM modules

Primary/recurrent cancer	Cancer type	Wider cancer groups	QoL measures
Primary	Colorectal	Colorectal	QLQ-CR29
	Anal	Anal	QLQ-ANL27
	Sarcoma	Sarcoma	N/A*
	Ovarian	Gynaecological	QLQ-OV28
	Cervical		QLQ-CX24
	Endometrial		QLQ-EN24
	Vulval		QLQ-VU34
	Prostate	Urological	QLQ-PR25
	Bladder		QLQ-BLM30
Recurrent	Colorectal	Colorectal	LRRC-QoL
	Anal	Anal	QLQ-ANL27
	Sarcoma	Sarcoma	N/A*
	Ovarian	Gynaecological	QLQ-OV28
	Cervical		QLQ-CX24
	Endometrial		QLQ-EN24
	Vulval		QLQ-VU34
	Prostate	Urological	QLQ-PR25
	Bladder		QLQ-BLM30

* no EORTC sarcoma-specific PROM existed at the time of the protocol development and therefore this group of patients will not fill out any additional cancer-type-specific PROM.

LRRC, Locally Recurrent Rectal Cancer; PROM, Patient Reported Outcome Measures; QoL, quality of life.

EQ-5D-5L^38^ is a well-established generic measure of HrQoL, with the added benefit of utility in generating incremental cost-effectiveness ratios between groups and quality-adjusted life years (QALYs). EORTC-QLQ-C30^39^ with specific cancer-type modules are validated cancer-specific PROMs. Decision regret questionnaire^41^ is a validated measure of the level of distress or remorse a person experiences after making a specific healthcare decision. Following a Patient and public involvement (PPI) engagement day, we have added the LRRC-QoL,^40^ a validated patient-reported outcome measure, capturing disease-specific QoL in locally recurrent cancer patients.

Patient health economic impacts, with financial and patient-important hidden toxicity measurements will be evaluated by:

- Calculating Index of Multiple Deprivation by using postcodes.
- Comprehensive Score for Financial Toxicity (COST) at all time points.^42^
- A bespoke patient employment and financial status questionnaire pre-surgery and 12 months.
- A patient reported health resource utilisation questionnaire at all post-surgery time points.

The COST measure^42^ is a validated PROM for financial toxicity for cancer patients. Additionally, quality-adjusted life years gained and incremental cost-effectiveness ratios between groups will also be assessed using EQ-5D-5L over 12 months after surgery.

Patients will undergo EMCS and perineal reconstruction, with either flap or biological mesh or skin closure reconstruction based on their treating surgeon’s preference. This is NHS standard of care, and no deviations or randomisation will occur. This data will be collected from surgical teams using their operation notes.

During the post-operative period, the Clavien-Dindo score,^43^ Comprehensive Complication Index^44^ and the EPS core outcome dataset^11^ will capture in-hospital complications, over 12 months after surgery.

NHS healthcare utilisation, cost-effectiveness and economic impacts according to National Schedule of NHS costing (Healthcare Resource Group - HRG codes) for each reconstruction type will be assessed. Of particular importance, in-hospital stay, critical care utilisation, theatre utilisation, further in-hospital procedures, reconstruction type, in-hospital complications, readmission, re-operation and morbidity up to 12 months after surgery will be collected and costed.

Complications are typically assessed in-hospital up to 90-days, but a 12-month follow-up will assess longer-term morbidity and its health economic impact. A 12-month follow-up balances time efficiency, with high participant study retention and response rates, as endorsed by our PPI group.

Patient demographics, cancer demographics, tumour and survival outcomes, along with any radiological recurrence data will be routinely collected up to 60 months after surgery as part of standard of care in all centres.

All data will be entered onto a bespoke, online, secure, patient-facing database (REDCap). The database will be centrally accessible, with patients receiving a unique anonymised study ID that will follow them throughout the whole study. This database is linked to NHS Spine (NHS GP records) that provides centrally monitored mortality follow-up.

### Outcome measures

#### Primary outcome

EORTC-QLQ-C30^39^ generic global health at 12 months after surgery. This validated QoL measure assesses physical, role, emotional, social and cognitive functioning domains, as well as global QoL and health, including three symptoms: fatigue, pain and nausea/vomiting. For all scales scores range from 0 to 100. For global and functional scales higher scores reflect favourable QoL, whereas higher symptom scales score indicates more symptoms.

#### Secondary outcomes

EORTC-QLQ-C30 generic and cancer specific module (https://qol.eortc.org/modules/) will be measured before surgery and at 3, 6 and 12 months after surgery. Additional validated cancer-specific modules will improve precision for various cancer types recruited. This approach allows for reliable between-group comparisons and longitudinal QoL trajectory assessments with bespoke QoL collected for each tumour subtype. All questionnaires can be administered in several languages.

EQ-5D-5L^38^ (https://euroqol.org/information-and-support/euroqol-instruments/eq-5d-5l/) will be measured before surgery and at 3, 6 and 12 months after surgery. This is a validated, National Institute for Health and Care Excellence (NICE) recommended measure of QoL encompassing five domains: mobility, self-care, usual activity, pain/discomfort and anxiety/depression.

Decision-Regret^41^ questionnaire is a validated 5-item scale measuring patient treatment-related decisions before surgery and at 3, 6 and 12 months after surgery.

LRRC-QoL^40^ will be measured before surgery and at 3, 6 and 12 months after surgery. It is a validated, locally recurrent rectal cancer patient-reported outcome. It consists of 9 multi-item scales (healthcare services, psychological impact, pain, urostomy-related symptoms, lower limb symptoms, stoma, sexual interest and urinary symptoms) and three single-item scales tailored for recurrent cancers.

Complications will be measured in-hospital and up to 12 months after surgery. The Clavien-Dindo score^43^ and Comprehensive Complication Index (CCI)^44^ are validated, gold-standard tools for reporting surgical complications. The CCI score increases measurement sensitivity and enhances standardisation in outcome reporting. The EPS dataset^11^ is an international Delphi-derived, core outcome dataset specifically assessing morbidity after EMCS and perineal reconstruction.

Health economic outcomes will include a comparison of costs and benefits, with financial toxicity measurements evaluated by the COST questionnaire^42^ measured before surgery and at 3, 6and 12 months. Quality-adjusted life years gained will be assessed using EQ-5D-5L (similar EQ-5D timepoints above).^45^ Healthcare resource use data will be collected, and unit costs from the National Schedule of NHS Costs will be used to cost resource use over the 12 months after surgery.^46^

Finally, overall and disease-free survival at 12 and 60 months after surgery will be measured.

### Sample size

The study is powered on the EORTC QLQ-C30. In accordance with published guidance, a mean difference of 10 points in the QLQ-C30 global health status score (0–100 scale) was considered to be consistent with the established minimally important difference between groups undergoing reconstructions at a ratio of 1:1 with mesh compared with flaps, with an SD of 25.^47 48^ This magnitude of difference was felt to be important to patients and clinicians and was recently used in a similar cohort study.

Assuming a statistical power of 80% and a two-sided type I error rate of 0.05, the total sample size required is 196 with 98 in each sample size group. Allowing for a 20% drop-out rate (accounting for failure to questionnaire return rate, balancing of groups, and patient deaths)—the final sample size required will be 236 with 118 in each group.

The perineal skin closure group will be analysed as a planned sub-analysis and will therefore be recruited in addition to the required sample size. This pragmatic approach was used as there is currently no validated patient reported outcome measure for this surgical population, nor an existing study that would enable a bespoke sample size to be performed.

As a real-world sense check, this sample size calculation was checked using data from the REMACS pilot phase (n=35) of patients who completed 6-month follow-up, mean QLQ-C30 global scores were 66.9 and SD 20.7. Including a 20% drop-out rate (<12% dropouts in pilot) the sample size required is 198 total. The overall predicted recruitment target of 236 will adequately power the study.

### Analyses

#### Statistical analyses

Descriptive statistics will be used to summarise demographic and clinical variables. For continuous variables, if data are normally distributed, mean (SD) will be calculated. If data are not normally distributed, the median (IQR) will be calculated.

Categorical/binary variables will be summarised as frequency and percentages of total. Data consistency and quality checks as specified in the data management plan will be undertaken. Statisticians will be blinded to reconstruction group until completion of analysis. Proportional differences will be assessed using the χ^2^ test or Fisher’s exact test, while continuous measurement differences will use the Student’s t-test or Mann-Whitney U test.

For the primary outcome, EORTC-QLQ-C30 global health scores at 12 months post-surgery will be compared between the flap and mesh reconstruction groups.

We acknowledge the importance of reducing allocation bias resulting from the observational design of this study, as the probability of being in a particular treatment group is not random (as in a randomised controlled trial), that is, some patients may be more likely to receive a certain treatment due to their characteristics and centre reconstruction preferences. We will therefore use propensity scoring to approximate randomisation methodology by balancing the patient characteristics in different treatment groups. As a first step, we will identify a set of variables which affect both the treatment and outcome (confounders) using directed acyclic graphs (DAGs) to visualise associations.^49^ We will include a combination of patient, disease and treatment factors, for example:

- Patient characteristics: age, sex, comorbidity, social deprivation.
- Disease characteristics: tumour site of origin, primary disease, recurrent disease, resection margin involvement.
- Treatment characteristics: radiotherapy dose, magnitude of surgery scored using the PE lexicon.^50^

We will also run a principal component analysis^51^ using the above clinical and demographic variables. This technique reduces the dataset into a smaller number of new variables (components), which can then be used to visualise patterns. The first two components will be displayed graphically to illustrate any clusters related to type of reconstruction. The second step will be to calculate a propensity score for each patient. This is defined as the conditional probability of assignment to a particular treatment given observed covariates. We will fit a multilevel logistic regression model in which patients are nested within surgeon within hospitals, and the outcome is treatment (ie, reconstruction method). Defined characteristics (with the potential to confound the outcome) are the independent variables.^52 53^ From this model, the predicted probability for each patient is the estimated propensity score. The distribution of propensity scores for flap and mesh reconstruction will be checked for overlap. If there are areas of no overlap, this indicates some patients (with a particular set of characteristics) have zero probability of receiving one of the interventions. If this is the case, we will look again at the covariates identified in DAGs in the first step.

We will also consider using principal component scores instead in the logistic regression model above. To do this, for all components with an eigenvalue>1, we will calculate a score for each patient based on each component’s coefficients. We will then use these to re-calculate propensity scores and re-check the distributions for overlap as above. If appropriate, we will consider removing subjects with extreme values of propensity scores.

The third step will be to balance the reconstruction groups by weighting by the inverse probability of treatment received (IPTW). ie,1 divided by the propensity score for mesh reconstruction, and 1 divided by 1 minus the propensity score for flap reconstruction.^51 54^ This weighting procedure will give more weight to those who had a low probability of mesh, and among those receiving flap, more weight will be given to those who were unlikely to receive flap, that is, the individuals under-represented in their treatment group. In order to check whether this weighting has balanced the covariates, a table and graph will be produced summarising unweighted and weighted standardised mean differences (SMD) between mesh and flap, with a value of greater than 0.100 taken to indicate significant imbalance.^55 56^ If there is significant imbalance, we will identify subjects with very large weighting and check their data/refine the propensity score model. If appropriate, we will consider truncating the weights so that all values of the weights larger than the 1st or 99th centile are replaced by that threshold value.

Finally, we will obtain an estimate of the IPTW average treatment effect by combining the multilevel propensity score weights with a regression model with EORTC-QLQ-C30 global health score at 12 months post-surgery as the outcome variable, adjusting for baseline score, and including cluster-robust standard errors. A planned subgroup analysis for primary skin closure group will be undertaken.

For secondary outcomes, the EQ-5D subscales and overall health utility score will be tabulated at each timepoint, with repeated measures performed using mixed modelling (overall health scale as the outcome variable, and baseline health scale, group, time and interaction between time and group as explanatory variables) to investigate the effect of reconstruction at all timepoints. Trajectories will be plotted from before surgery to 12 months.^29^ This will be repeated for the EORTC-QLQ-C30 global, cancer-specific scores, LRRC-QoL and Decision-Regret scores with mixed effects ordered logistic regression considered for analysis of subscales investigating changes over time for all reconstruction groups. These plots will be used in future work to develop a decision tool with patients.

The Clavien-Dindo complication score is an ordinal variable, the Wilcoxon-Mann-Whitney test will be used to compare morbidity between groups. If appropriate, ordinal logistic regression will be used to perform an analysis adjusted for important prognostic variables.

The CCI score over a 12-month observational period will be checked for normality, summarised and compared between groups, either using a t-test or Wilcoxon-Mann-Whitney test. If appropriate, a multiple regression model adjusted for baseline will be performed.

Recurrence and survival (overall and disease-specific) analyses will be reported using Kaplan-Meier analysis and compared using weighted log-rank test and Cox proportional hazards models.

#### Health economic analyses

We conducted a targeted review of the literature, searching for cost-effectiveness models for PE to inform the design of our analysis.^33 57 58^

A prospective, within-study, real-world, cost-utility economic analyses, as per NICE recommendations,^59^ will be conducted comparing reconstruction techniques. The economic analysis will use the model structure, previously used in our earlier study.^35^ The analysis will first be completed from an NHS and Personal Social Services perspective, and then a scenario analysis using a societal perspective including patients financial toxicity costs determined using COST will be conducted.^42^ A bespoke questionnaire that will quantify loss of earnings or employment will be used. The time horizon of the analysis will be 12 months, but we will conduct scenario analyses using a longer time horizon beyond the trial. The analysis will not include discounting for the 12-month time horizon but for scenarios with longer time horizons, annual 3.5% discounting will be used. The model will have 3-month cycles to align with collection of quality-of-life data and other patient outcomes.

Healthcare utilisation data will be prospectively collected on REDCap. Data will be collected for each patient and their method of reconstruction, accounting for in-hospital stay, critical care utilisation, theatre utilisation, additional in-hospital procedures, in-hospital complications, hospital readmission, follow-up costs and treating consequential complications over the 12 months after surgery.

Unit costs for healthcare resource use will be taken from standard published sources such as NHS Reference costs^60^ and Personal Social Services Research Unit,^46^ and, if necessary, micro-costing from the local hospital finance unit. Longitudinal EQ-5D-5L QoL data will be converted into EQ-5D 3L values using the mapping algorithm recommended by NICE, as applicable and according to latest guidance. The economic model will also compare patient overall survival. Models will be conducted as per NICE guidelines and best practice.^60^ Results will be presented as an incremental cost-effectiveness ratio (incremental cost per QALY gained). We will investigate uncertainty around the analysis results by conducting probabilistic sensitivity analyses and uncertainties relating to model structure or choice of data sources/inputs through scenario analyses.

#### Data collection—qualitative study

This embedded qualitative study aims to:

1. Describe patient experiences over time, focusing on preparedness, decision-making, recovery, well-being and hidden toxicities after EMCS surgery.
2. Characterise the patient experience and impacts of different cancer treatment options (surgery, chemoradiotherapy, reconstruction type), perineal complications and decisional regret.
3. Identify the resources and support needed by patients to prepare for, recover from or manage the impact of EMCS.

### Sampling

Participants will be recruited from the observational cohort study, adhering to the same inclusion and exclusion criteria. Patients who meet purposive sampling criteria and have expressed an interest in qualitative interviews will be approached and consented separately (see *Recruitment and Consenting* section).

Up to 30 patients will be purposefully sampled from participating centres for virtual interviews via MS Teams or telephone. Given the paucity of qualitative data in this field, this sample size will be sufficient to obtain information power.^61 62^ Sampling will ensure diverse representation from UK centres, balancing cancer characteristics, reconstruction methods and maximising ethnic, social and economic diversity. Priority will be given to young cancer survivors (<45 years), patients encountering EPS complications, individuals of Black or Southeast Asian ethnicity and patients with rare cancers (ie, anal cancer and sarcomas). Demographic observational study data will inform qualitative study sampling. Drop-outs will be managed according to mitigating strategies below.

We will actively approach patients for whom English is not their first language, from under-represented ethnic minorities and lower socioeconomic groups, who may present with more advanced cancers due to health access inequality. Experiences captured from these groups are critical. We will work with our charity partners ensuring representation of hard-to-reach populations, those with rare tumour types and patients who are not digitally connected. This will maximise equality, diversity and representation of people who ‘are seldom asked’.

Semi-structured, 60 min interviews will be conducted at 3 and 12 months after surgery, with flexibility to conduct them up to 21 days before or after the set time point depending on the patient’s availability and convenience. Patients will receive digital or paper invitation packs before the 3-month time point. They will be re-consented for interviews and contacted by the research team to arrange a suitable interview date. Participants will be advised of the second interview 8 months later, with their status checked with the recruiting site and consent checked with the patient, before proceeding to the second interview. Interviews will be conducted by an experienced qualitative researcher virtually or over the telephone depending on the patient’s preferences, recorded on an encrypted audio recorder and translator services will be available, ensuring equity of access.

Interviews will follow an open-ended schedule. The topic guide was co-developed with PPI during the study pilot, with details given in online supplemental appendix 1. This allows for interview flexibility, with pilot phase feedback indicating that patients volunteered additional information that was not initially captured. Questions around the feeling of losing part of oneself, coping inability, body image changes and ‘scanxiety’ were subsequently added. Patients had no issues discussing sensitive topics and valued the interviews taking place at the two proposed post-surgery timepoints because meaningful discussions were undertaken with the benefit of experience after complications and in-hospital recovery. The semi-structured nature of the interviews means that patients will be able to share their stories in their own way, guided by the interviewer in the form of prompts. Interviews will follow a temporal trajectory with patients invited to describe the factors leading to them being offered surgery, followed by their preparation for and decision-making processes. They will then be asked to reflect on their experiences of the surgical procedure and recovery, including their current circumstances at 3 months post-surgery.

### Qualitative analysis

During and immediately after each interview, the interviewer will record reflexive notes. Audio transcription will be transcribed verbatim, checked against the recording and anonymised. Data will be uploaded to NVivo for data management. Preliminary summaries will be written after each interview to identify emerging themes to follow-up discussions. The follow-up interview will ask patients to reflect on the content of their previous interview and discuss any changes.

Longitudinal interview analyses will use constant comparative methods from grounded theory. The data will be coded using NVivo’s framework matrix facility to examine themes longitudinally, enabling comparisons within each case and across cases, focusing on changes over time.

The stages of data analysis, drawing on longitudinal comparisons, will include:

1. Initial reading: Review interview data and record any initial reflections.
2. Preliminary coding: Two researchers code the data.
3. Team meeting: Discuss and refine codes with wider research/PPI team.
4. Individual case coding: Code all interview data for each participant.
5. Categorising codes: Group codes into categories for each case.
6. Longitudinal comparisons: Within case and category comparisons focusing on changes over time.
7. Focused coding: Examine categories in relation to emerging concepts and phases.
8. Discussion on themes: PPI co-led focus group discussions of final themes.
9. Development and dissemination: Report write-up and dissemination.

Based on an iterative process, emerging themes will be used to develop explanatory accounts. Analysis will draw on sociological perspectives of illness adaptation, recovery and self-management in addition to psychological theories of preparedness, flexibility, coping and individual behaviour change.

## Patient and public involvement statement

REMACS was co-designed with patient, public and charity partners. The study design including the observational, health economic and qualitative elements has been designed with patients. PPI has been embedded throughout the development and conduct of the REMACS study. Patients with lived experience of EMCS, representatives from cancer charities and public contributors were involved from the initial conception of the study, identification of research priorities, study design, funding applications and selection of outcome measures. They are also included as co-applicants on the funded trial and as co-authors on this manuscript.

A dedicated PPI advisory group, including representatives from Bowel Research UK, PLANETS Cancer Charity, Communities First and patient partners with lived experience of advanced and recurrent pelvic cancers, contributed to defining the research questions and ensuring that the study addresses issues of greatest importance to patients living with and beyond cancer. PPI contributors highlighted the need to understand longer-term quality of life, decision regret, financial toxicity, hidden treatment toxicities and recovery following perineal reconstruction. Their input directly informed the selection of patient-reported outcome measures and the inclusion of health-economic and decision-regret outcomes.

During protocol development, feedback from a PPI engagement event resulted in the addition of the Locally Recurrent Rectal Cancer Quality of Life (LRRC-QoL) questionnaire to better capture disease-specific concerns. PPI contributors also co-developed the qualitative interview topic guide and identified important areas for exploration, including body image, loss of identity, coping difficulties, fear of recurrence (‘scanxiety’), preparedness for surgery and the impact of complications on everyday life.

PPI representatives continue to contribute to study governance through membership of the study management group and are named collaborators on the funding application. They will advise on recruitment strategies, participant-facing materials, interpretation of findings and dissemination activities. The planned 12-month follow-up period was supported by PPI contributors, who considered this duration appropriate to capture meaningful recovery and survivorship outcomes while maintaining participant engagement.

To maximise inclusivity and representation, PPI partners have advised on approaches to engage people who are seldom heard in research, including individuals from ethnic minority backgrounds, socioeconomically disadvantaged communities, younger cancer survivors and those with rare tumour types. Their recommendations have informed the purposive sampling strategy for the qualitative component and the use of flexible methods of questionnaire completion and interview participation.

Study findings will be disseminated in partnership with PPI contributors through patient organisations, charity networks, public engagement events, social media and lay summaries. PPI representatives will also contribute to the interpretation of qualitative findings and co-development of future patient decision-support resources arising from this work.

## Ethics and dissemination

Ethics approval for the study has been granted by Health Research Authority Research Ethics Committee (22/NE/0032). Registration with clinicatrials.gov (NCT05219058) is also complete.

Once the study is complete results will be disseminated via scientific publication and presentation at meetings. The findings will also be disseminated to researchers, healthcare professionals, patients, carers, charities and policymakers. This will be delivered through online platforms, media and engagement events with the support of our dedicated PPI advisory group and charity collaborators. We aim to raise awareness of late effects of cancer treatment, influence international guidelines and cancer commissioning.

Beyond this study we intend to use the findings to develop a patient decision aid for use in EMCS shared decision-making consultations. This will be undertaken as an implementation study and validated through mixed methodology.

## Supplementary material

10.1136/bmjopen-2026-124218 online supplemental appendix 1
