# Supplementary material for: Understanding the impact of perineal reconstruction after extended margin cancer surgery on longer-term quality of life, morbidity and health economic outcomes: protocol for the UK REMACS observational cohort study
Source: BMJ Open. 2026 Sep 29;16(9):e124218. doi: 10.1136/bmjopen-2026-124218 (PMC13630098; doi:10.1136/bmjopen-2026-124218)
Supplement: Supplementary file 1 [file bmjopen-2026-124218.reviewer_comments.pdf]

PEER REVIEW HISTORY

BMJ Open publishes all reviews undertaken for accepted manuscripts. Reviewers are asked to complete a checklist review form and are provided with free text boxes to elaborate on their assessment. These free text comments are reproduced below.

ARTICLE DETAILS

Title (Provisional)

Understanding the impact of perineal Reconstruction after Extended Margin Cancer Surgery on longer-term quality of life, morbidity and health economic outcomes: Protocol for the United Kingdom REMACS observational cohort study

Authors

West, Malcolm A.; Khan, Kashuf; West, Charles Timothy; Li, Zoe; Brown, Elias; Sodergren, Sam; Moyses, H. E.; Rose, Sam A; Bignall, Errol; Stephen, Layla; Applin, Sharon A; Cooper, Keith; Souto Riberio, Inês; Calman, Lynn; Mirnezami, Alex H

VERSION 1 - REVIEW

**Reviewer**	**1**
**Name**	**Sohail, Amir H.**
**Affiliation**	**NYU Langone Health**
**Date**	**17-Jul-2026**
**COI**	**None**

Well written manuscript describing the study protocol

**Reviewer**	**2**
**Name**	**Komori, Koji**
**Affiliation**	**Aichi Cancer Center**
**Date**	**07-Aug-2026**
**COI**	**None**

This paper is excellent. It is acceptable for acceptance as it stands.

VERSION 1 - AUTHOR RESPONSE

Thank you for your review.

We are glad that there are no reviews requested from either of the reviewers.
