# Supplementary material for: Understanding the impact of perineal reconstruction after extended margin cancer surgery on longer-term quality of life, morbidity and health economic outcomes: protocol for the UK REMACS observational cohort study
Source: BMJ Open. 2026 Sep 29;16(9):e124218. doi: 10.1136/bmjopen-2026-124218 (PMC13630098; doi:10.1136/bmjopen-2026-124218)
Supplement: Supplementary file 2 [file bmjopen-16-9-s001.docx]

**Appendix 1**

Topic guide and sample interview questions at 3-months and 12-months after surgery

**Questions:**

1. Can you please tell me about your experiences of having the operation?

*(Rapport building)*

*Follow-up questions:*

*What led you to have the operation?*

*What was the operation like?*

*What was your recovery like?*

2. Can you tell me about any post-operative complications you experienced?

*Short and long-term effects*

3. What is life like for you now as a result of these complications? Could you describe the limitations you have experienced as a result of these complications?

Prompts: Problems sitting and laying down

a. Mobility
b. Daily activities
c. Working
d. intimate relationships

4. How do you feel about these complications?

*(How did you cope with these complications?*

*Have you found ways to adapt to these complications? (New normal)*

5. Some patients have indicated that after this big operation they may still get sensations from areas removed during surgery – what experiences, if any, have you had like this?

*(Loss of part of yourself)*

a. Feelings of needing to open your bowels or pass urine *(autonomic)*
b. Altered feelings in the sensation and muscles between your legs *(somatic)*
c. Feeling part of yourself is lost and awareness something is missing *(psychological)*

6. Some patients we have previously spoken with have mentioned feeling traumatised by their experiences and referred to this as post-traumatic stress. Is this something you recognise?

*What did that feel like for you? How did you cope with this?*

7. Tell me about how your understanding of what was happening to you impacted on your experiences?

*(Knowledge facilitates coping and not knowing complicates coping)*

a. How knowledge helped coping
b. How not knowing made coping more difficult

8. How did this experience impact on your overall recovery to get back to normal after surgery?

*(Rollercoaster to the new normal / Complications as bumps in the road / No control)*

a. Comparison of complications to other post-surgery hurdles
b. Lack of control

9. Your doctors will have used some reconstructive techniques at the end of the surgery to help put your body back together again, how has this reconstruction impacted on your recovery?

*(Morbidity from reconstruction - explore overall thesis research question)*

a. Complications (Flap morbidity / flap donor site morbidity / mesh problems)
b. Body image (Change in appearance / animal derived foreign body)
c. Choice of reconstruction

10. In light of your experiences and the complications you experienced how do you feel now about the decision to go ahead with surgery for the dangerous tumour that you had?

*(Journey is worth it / Life is threatened / Forced to accept risks)*

a. Future perspective
b. Positive feelings / regret
c. Shared-decision making

11. If you could speak to yourself before experiencing this complication, what would you say to make it easier? What would you say to others who are going through a similar experience?

*(Survivors’ insight supports coping)*

a. Useful information
b. Useful / adaptive actions

12. Is there anything you wanted to talk about which I did not ask you about?
